# Do Stress Trajectories Predict Mortality in Older Men? Longitudinal Findings from the VA Normative Aging Study

**DOI:** 10.4061/2011/896109

**Published:** 2011-09-27

**Authors:** Carolyn M. Aldwin, Nuoo-Ting Molitor, Spiro Avron, Michael R. Levenson, John Molitor, Heidi Igarashi

**Affiliations:** ^1^Human Development & Family Sciences, School of Social & Behavioral Health Sciences, College of Public Health & Human Sciences, Oregon State University, Milam Hall, Corvallis, OR 97331, USA; ^2^Department of Epidemiology and Public Health, Imperial College School of Medicine, Norfolk Place, London W2 1PG, UK; ^3^Normative Aging Study, VA Boston Healthcare System (151MAV), 150 South Huntington Avenue, Boston, MA 02130, USA; ^4^Departments of Epidemiology and Psychiatry, Boston University Schools of Public Health and Medicine, 715 Albany Street T 3E, Boston, MA 02118, USA

## Abstract

We examined long-term patterns of stressful life events (SLE) and their impact on mortality contrasting two theoretical models: allostatic load (linear relationship) and hormesis (inverted U relationship) in 1443 NAS men (aged 41–87 in 1985; *M* = 60.30, SD = 7.3) with at least two reports of SLEs over 18 years (total observations = 7,634). Using a zero-inflated Poisson growth mixture model, we identified four patterns of SLE trajectories, three showing linear decreases over time with low, medium, and high intercepts, respectively, and one an inverted U, peaking at age 70. Repeating the analysis omitting two health-related SLEs yielded only the first three linear patterns. Compared to the low-stress group, both the moderate and the high-stress groups showed excess mortality, controlling for demographics and health behavior habits, HRs = 1.42 and 1.37, *p*s <.01 and <.05. The relationship between stress trajectories and mortality was complex and not easily explained by either theoretical model.

## 1. Introduction

A lifespan developmental approach to stress and health is based on two premises. First, stressors are not isolated occurrences but can have lifelong effects on health through setting up different patterns of vulnerability and resilience [1, 2]. The second premise is that there are increasing individual differences in stress and health trajectories with age [3]. However, there are surprisingly few longitudinal studies of changes in stress, especially in later life. Inconsistent results in the literature on the effects of stress on health and mortality may reflect the aggregation of individuals who may have had very different experiences over their life. 

The study had two purposes. First, using 18 years of data in a large sample of middle-aged and older men, we sought to identify patterns of change in stressful life events across the lifespan. The second was to examine the effects of different types of stress trajectories on mortality. Specifically, we contrasted an allostatic load model [4] which assumes that higher levels of chronic stress will have the greatest adverse effects on health, with a hormesis model [5–7], which suggests that moderate amount of stress may have protective effects on health.

### 1.1. Aging and Stressful Life Events

Early correlational studies found that the number of life events is negatively associated with age (for reviews see [8, 9]). However, most life events inventories sample events more common in young adulthood, such as graduations, new jobs, marriages, having children, and divorces. Newer life event measures that targeted middle-aged and older adults show little or slightly negative correlations with age [8] although health-related events show increased frequency in late life [9], as do loss events [10, 11]. A few longitudinal studies have examined age-related changes in the number and type of stressful life events.

Chiriboga [12] found that young adults reported more life events (both negative and positive) than older adults, but there were no differences between individuals in mid-life and late life. However, the longitudinal analyses spanning a 12-year period did not find a linear decrease with age. Rather, the number of events fluctuated over time, suggesting both cohort and period effects. In a preliminary six-year longitudinal study, Yancura et al. [13] found suggestions of nonlinear change over time, with the number of self-reported life events increasing until about age 65 and then decreasing into later life. Ensel and Lin [14, 15] used data spanning 15 years, but the 12-year gap between the last two assessments did not permit an examination of trajectories over time, although they did find that the more distal events predicted the more recent ones A few studies examined growth curve stress trajectories in adults [10, 11, 16] but over short periods of time (e.g., three to seven years). The results varied by type of stressor, the context, and respondent characteristics. For example, there was little change in stress over a three-year period among married women in their thirties, but recently divorced women had higher levels of stress that decreased over three years. Divorced women with antisocial behavior patterns decreased less, however. In older adults, especially in African Americans, loss-related events tended to increase [10, 11].

### 1.2. Stressful Life Events and Mortality

The adverse effects of stress on both mental and physical health are now well documented [17–20]. However, there are inconsistent results in the literature on the relationship between stress and mortality. While some studies have found that high levels of stressful life events do predict mortality [21, 22], this may be restricted primarily to health-related stressful life events [23]. Other studies have found no relationships between life events and mortality [24], and one found an inverse association [25]. 

Others have suggested that chronic stress may be a better predictor of health outcomes than the simple occurrence of a life event [26, 27]. However, even in this area, there are mixed results. For example, while Schulz and Beach [28] showed an increased risk of mortality among caregivers, Fredman et al. [29] found a decreased risk—perhaps because those who are healthier are more likely to take on the arduous task of caregiving. 

Allostatic load (AL; [30]) is a construct assessed across multiple biosystems, including cardiovascular, metabolic, neuroendocrine, and inflammatory markers that are presumed to reflect chronic psychosocial stress. A number of studies have shown that AL is associated with higher mortality levels (for a review, see [31]). However, few studies have examined psychosocial stress measures in the context of allostatic load, and the ones that have generally find little or no relationship between various components of AL and psychosocial stress [32]. Yancura et al. [33] found that stress was unrelated to a similar measure, the metabolic syndrome, although positive coping strategies were protective. 

There are a number of possible reasons for the difficulties in relating stress to mortality. In addition to individual differences in vulnerability to stress, such as personality, coping, and differential access to social resources, very few studies actually examine stress over a long period of time. Given that the types of chronic illnesses related to mortality often take decades to develop, it would be surprising if a single assessment of life events were strongly associated with mortality. Even the chronic stress measures are seldom longitudinal. Thus, examining long-term stress patterns may be a more promising way to link stress with mortality.

However, there is another possible explanation. While most stress theories implicitly assume that there is a linear relationship between stress and adverse outcomes, there is some evidence to suggest that there is a nonlinear relationship. Biogerontologists have recently become interested in *hormesis, *which is defined as “the phenomenon in which adaptive responses to low doses of otherwise harmful conditions improve the functional ability of cells and organisms” ([34], page 1). Most researchers assume that there is a linear, dose-response relationship between stress and adverse health outcomes, but hormesis suggests that there is a nonlinear or inverted U relationship, such that low or even moderate doses of an otherwise toxic substance provide protective benefits against future exposure to stresses. Although still controversial, there is a growing body of evidence for hormesis in organisms such as flatworms, plants, and mice [6, 7, 35], as well as in cardiac and neuronal cells [36]. Presumably, the mechanism is the activation of stress repair mechanisms at the cellular level, such as heat shock proteins. While hormesis has been studied most frequently in plant and animal models, it has been linked to longevity in humans [35, 37]. However, the study of hormesis typically involves physical stressors such as radiation, and little work has been done with psychosocial stressors. 

Dienstbier's [38] construct of “physiological toughening”—in which intermittent exposure to stress results in better functioning—is consistent with the hormesis model. The field of stress-related growth [39] has also challenged the assumption of linear effects of stress on health psychological outcomes. Applying the models to human stress and health leads to some interesting and somewhat counterintuitive hypotheses, namely, individuals who experience intermittent or moderate amounts of stress may develop better coping resources which allow them to be more resilient to stress in later life. For example, in a longitudinal study, Schnurr et al. [40] found that individuals who experienced moderate combat exposure had improved MMPI profiles 20 years later, but individuals with no or high combat exposure had worsened MMPI profiles. 

People who have chronically high stress levels may experience resource depletion [41], leading to impaired ability to cope with stress. However, individuals who, for whatever reasons, avoid stressors, that is, who are extremely risk averse, may not develop the resources which allow them to adequately cope with problems that arise in late life. In a qualitative study, Vaillant [42], described one such person who managed to adopt an extremely self-limiting lifestyle in that she managed to avoid employment, marriage, and even friends, but was poorly equipped to deal with problems in later life. 

A possible explanation for the inconsistent relations between stress and mortality is that hormesis may apply to psychosocial as well as physiological stressors. Thus, the possibility exists that there is a nonlinear relationship between stress and mortality—that moderate stress levels may be associated with better longevity, while both chronic high and low levels may be associated with poorer longevity.

### 1.3. Present Study

The primary aim of this study was to compare longitudinal change in life events and its consequences on mortality, using 18 years of data from the VA Normative Aging Study (NAS), a longitudinal panel study which has followed a large sample of men since the mid-1960s. The NAS has collected up to nine assessments of stressful life events. To our knowledge, this constitutes the longest extant longitudinal study of stress and coping using quantitative measures and, as such, provides a unique resource to model changes in stress patterns from mid- to late life and their relationships to mortality.

First, we examined the developmental trajectories of stressful life events. Only a few studies have examined trajectories of life events in adulthood [10, 11, 16]. We hypothesized that life events would increase until approximately age 65 and decrease thereafter [13]. Further, we hypothesized that by using latent class growth analyses (LCGAs; [43]), we would identify at least three different patterns of trajectories, with some individuals exhibiting chronically elevated levels of life events, others showing stable low levels, and others showing moderate or intermittent levels of life events. We estimated two sets of LCGA models, one for stressful life events measure including health items, and one for a measure excluding health items. The purpose of the first set of analyses was to examine how stressful life events, including health problems, changed with age; the second omitted health problems in order to avoid a potential confound between stress and health when predicting mortality. 

The second aim was to understand how these developmental trajectories of stress related to all-cause mortality, contrasting an allostatic load model with a hormetic one. If the allostatic load model is correct, there should be a linear relationship between stress and mortality. However, if the hormesis model is correct, then the relationship should be nonlinear (U-shaped, with higher mortality at either end of stress distribution). We hypothesized that individuals in the chronic high- or low-stress categories would be more likely to have higher rates of mortality than those in the intermediate stress categories.

## 2. Methods

### 2.1. Sample

The VA Normative Aging Study (NAS) screened over six thousand men between 1961 and 1968 and enrolled 2,280, aged 21–81, who had good health, defined as the absence of chronic illness and blood pressure below 140/90 as well as likely geographic stability, which was indexed by extensive ties in the Boston area (see [44]). The men are equally divided between blue and white collar workers, and reflected the racial profile of Boston in the late 1950s, that is, primarily white. As of August 2010, 852 (37.4%) were participating, 1,305 (57.2%) were deceased, 89 (3.9%) were lost or dropped out, and 34 (1.5%) were too sick to participate. The current mean age is 79 (SD = 6.4, range = 63–98). 

In this study, we began with 1,565 men who completed the initial stress inventory in 1985. We computed stress trajectories from 1985 to 2002 (an 18-year period) on the 1443 men who had at least three assessments of stressful life events. Mean age of this sample in 1985 was 60.30 (SD = 7.73, range = 41–87). We then predicted subsequent mortality, using these stress trajectories as a predictor, and including covariates from various surveys and medical examinations, which decreased the *n* to 977. This subsample did not differ from the larger sample in health ratings, smoking, alcohol consumption, or education although they were more likely to be married, *X* ^2^(1; *N* = 1222) = 4.30, *p* < .05.

### 2.2. Procedures

NAS men complete biomedical examinations every three years as well as periodic mail surveys assessing psychosocial variables, which typically have response rates exceeding 80%. The NAS began collecting stressful life events in 1985, using three mail surveys over six years, which also included information on mental and physical health. Response rates for these surveys were typically over 80%. In 1987, the Health and Social Behavior (HSB) survey was developed to obtain psychosocial information at the time of the biomedical exam. The HSB was included in a packet of materials sent to the men before they reported for their triennial physical exam. The HSB included measures assessing stress and mental and physical health; response rates typically exceed 95%. Data collected between 1985 and 2002 were selected to develop the stressful life event trajectories. Data from the triennial medical exams and several surveys were used for the covariates.

### 2.3. Measures

#### 2.3.1. Stressful Life Events

In the NAS, the Elders Life Stress Inventory (ELSI; [8]) was included in both the Social Support and the Health and Social Behavior (HSB) surveys. The ELSI is a 30 item measure that assesses events likely to occur in middle-aged and older adults during the past year. These items include institutionalization of parent or spouse, child's divorce and retirement. The ELSI uses a nested construction technique; that is, where possible, there are several items in each domain such that the more items that are reported, the more serious are the problems in that domain. For example, the disruptions in marital relationships domain contains three items (e.g., marital problems, separation, and divorce). Thus, the stress ratings correlate .96 with simple counts of items. For those analyses with health outcomes, scoring for the ELSI excludes the items related to worsening health. The ELSI has good criterion validity, correlating about .2 to .3 with physical and psychological health outcomes, comparable to other stressful life event measures. Although this life events measure is correlated with other types of stress measures, such as hassles and perceived stress, as well as personality measures such as neuroticism, it contributes independent variance to health outcomes [45]. 

We scored the ELSI in two ways: a total sum which includes all 30 items and one which omits the two items which tap health events. As mentioned earlier, we used stressful life events from 1985 to 2002. We used a total of 7,634 observations for 1443 men (*M* = 5.29, SD = 1.58, range = 3–9).

#### 2.3.2. Covariates

In the models predicting mortality as a function of stress trajectory, we included a number of covariates that are known to affect mortality, including a self-reported health rating, demographics, and health behavior habits. 

Educational attainment was derived from an item in the original Social Screening Survey which was collected at study enrollment. This was recorded into less than high school (9.31%), high school (24.97%) least some college (51.18%), and graduate or professional education (14.53%). Marital status was derived from a survey in 1986 and dichotomized into married (88.46%) versus not married (11.54%).

For self-reported health, we used a single item assessing general health from the first Social Survey in 1985 taken from the SF-36 [46]. Health was rated from 1–5, where 1 = poor and 5 = excellent (*M* = 4.2, SD = 0.72). Note that 90% rated their health as good or excellent. Drinking status was derived from a survey item administered in 1986 and was coded to identify nondrinker (12.93%), light or moderate (71.69%), and heavy drinkers (15.38%). Current smokers (13.03%) were identified based on an interview at the physical exam closest to 1985.

#### 2.3.3. Mortality

Vital status of NAS participants was monitored by periodic mailings, and when notified of a death, official death certificates were obtained. Death certificates were reviewed by a physician, and cause of death coded by an experienced RN using ICD-9.

### 2.4. Analyses

To identify different trajectory classes of stressful life events, we used a semi-parametric mixture-model approach for Nagin [47] and Jones et al. [43]. The method is implemented in the TRAJ procedure in SAS [43] and uses a group-modeling approach that employs a mixture of probability distributions that are specified to match the data being analyzed. The mixture models used deal with heterogeneity in the population by modeling subpopulations which differ in regard to their parameter values. The marginal density for the outcome *y* is

(1) $$f\left(y\right)=\overset{K}{\underset{k=1}{\sum }}\text{Pr}\;\left(C=k\right)\text{Pr}\;\left(Y=y\;|\;C=k\right)=\overset{K}{\underset{k=1}{\sum }}p_{k}f\left(y,\lambda_{k}\right),$$

where *p*_*k*_ the probability of belonging to class *k* corresponding to parameters *λ*_*k*_, which depend on time, thus creating longitudinally based latent trajectories. 

**Figure 1:**
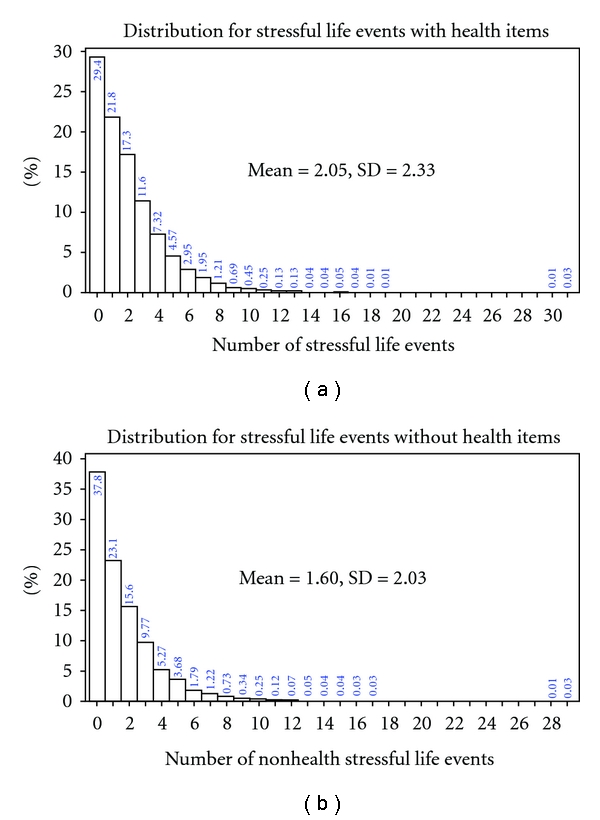
Distribution of the stressful life event measures.

The TRAJ procedure allows one to model Pr (**Y** = *y* | *C* = *k*) in a number of ways, including a standard Poisson model appropriate for count data. However, because approximately 30% of NAS men did not report stressful life events (see Figure 1), it was necessary to use a zero-inflated Poisson (ZIP) model to account for the fact that there are more zeros than would be expect under the Poisson assumption [48]. As with standard Poisson models, covariates such as age are related linearly to the log of the Poisson mean. We used the Voung [49] test to compare the zero-inflated model with an ordinary Poisson regression model for both the SLE sum and the SLE sum omitting the health items. In both instances, the Vuong Non-Nested Hypothesis test statistics suggested the ZIP model was superior to the standard Poisson model (*p* < .001 for both outcomes).

We computed trajectories of life events against age centered at 63. In addition, we tested a quadratic term for age, based on preliminary analyses, which suggested that there might be nonlinear trajectories [13]. The number of classes present in the data is determined by comparing models with the same class structure but varying numbers of classes. In order to identify the correct number of classes, we used the change in Bayesian information criterion (BIC, [50]) between models as an approximation to the log of Bayes factor [51], 2log _e_ (B_10_) ≈ 2(ΔBIC), where ΔBIC is the BIC of the more complex (alternative) model minus the BIC of the simpler (null) model [52]. Finally, we considered the model's interpretability. 

In order to graph the predicted trajectories for each group, *c*, we used the following model, written with no covariates adjustments, which is based on the growth mixture-model described earlier in this paper:

(2) $$\begin{array}{cc} \varphi_{cj} & =p_{0cj}+\left(1-p_{0cj}\right)*p_{1cj},\\ p_{0cj} & =\frac{\exp\;\;\left(\alpha_{0c}+\alpha_{1c}*{\text{age}}_{j}\right)}{1+\exp\;\;\left(\alpha_{0c}+\alpha_{1c}*{\text{age}}_{j}\right)},\\ p_{1cj} & =\exp\;\;\left(\beta_{0c}+\beta_{1c}*{\text{age}}_{j}\;+\beta_{2c}*{\text{age}}_{j}^{2}\right),\; \end{array}$$

where *φ*_*cj*_ indicates the predicted probability for the group *c* at time *j* and *φ*_*cj*_  was composed using both predicted probabilities *p*_0*cj*_ and *p*_1*cj*_ which were obtained from the zero-inflation and the Poisson parts of the model. Note that parameters *α*_0*c*_ and *α*_1*c*_ are associated with the zero-inflation part of the model, while *β*_0*c*_, *β*_1*c*_, and *β*_2*c*_ are coefficients associated with the Poisson part of the model. Here, the estimate *β*_2*c*_ will be zero for groups where the nonlinear pattern is not best described with the group trajectory.

#### 2.4.1. Proportional Hazards Models

A Cox [53] proportional hazard model was used to investigate the effect of stressful life event trajectory classes on mortality. The general equation is

(3) $$\lambda \left(t_{i}\;|\;C_{i},X_{i}\right)=\lambda_{0}\left(t_{i}\right)e^{\beta_{1}C_{i}+\beta_{2}X_{i}},$$

where *λ*_0_(*t*_*i*_) is individual *i*'s risk of dying with baseline hazard rate (*λ*_0_) at time *t*, *C* is the class indicator estimate obtained from the growth mixture model in the first stage, and *X* represents the vectors of demographics and health behaviors. Parameter *β*_1_ represents the effect of the class of stressful life event trajectories on risk of dying, and parameter *β*_2_ represents the effect of demographics (i.e., education) and health behaviors (i.e, drinking) on risk of dying.

 Following Mroczek et al. [54], we used two different proportional hazard models, where the first model used only class assignments, and the second model utilized both class estimates and covariates to determine whether any of the effects of life events class on mortality were affected by the covariates.

## 3. Results

### 3.1. Types of Life Event Trajectories

An iterative procedure was used to determine the number of classes, with the number of classes determined by change in the model fit criterion, BIC [43]. We first examined the total life events measure, which included the two health items. As can be seen in Table 1, the fit improved as classes were added to the model, starting with a single-class model and moving up to a model with four classes. However, beyond four classes, the fit became worse. Thus, we determined that the four-class model provided the best estimate of the true number of classes.

Table 2 provides the intercept and linear slope estimates for the four classes. While we tested the quadratic term for all of the classes, it was only significant for the first class. We compared a simpler model in which only the first class had a quadratic term to a more complex model which estimated quadratic terms for all classes. The model comparison test for the more complex model did not significantly improve over the simpler model, 2ΔBIC = −14.23, so we retained the simpler model. 

**Figure 2:**
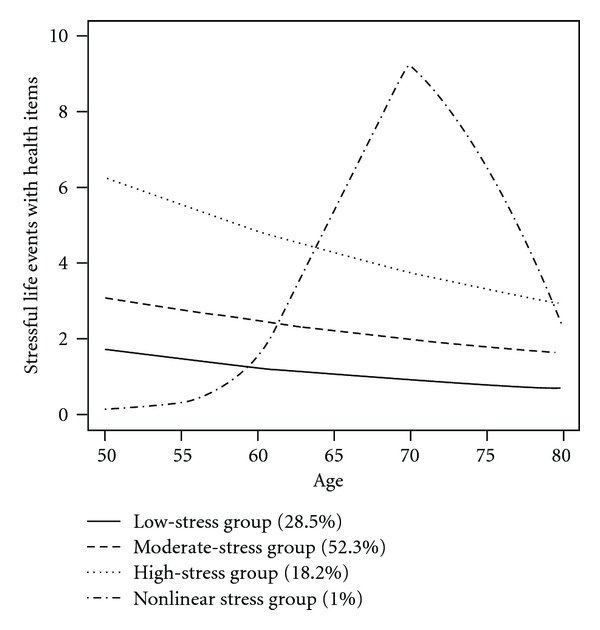
Graphs of the predicted trajectories for the different stressful life event classes (including health items).

Figure 2 presents the estimated trajectories for each of the four classes. Three of the classes showed decreasing slopes, with low, medium, and high intercept values. Class 1 (low stress), Class 2 (moderate stress) and Class 3 (high stress) contained 28.5%, 52.3%, and 18.2% of the sample. However, Class 4 (nonlinear) shows an inverted U-shaped relationship between life events and age, with life events increasing from age 50 to about 70, and decreasing thereafter. However, this group only contained about 1% of the sample. 

**Figure 3:**
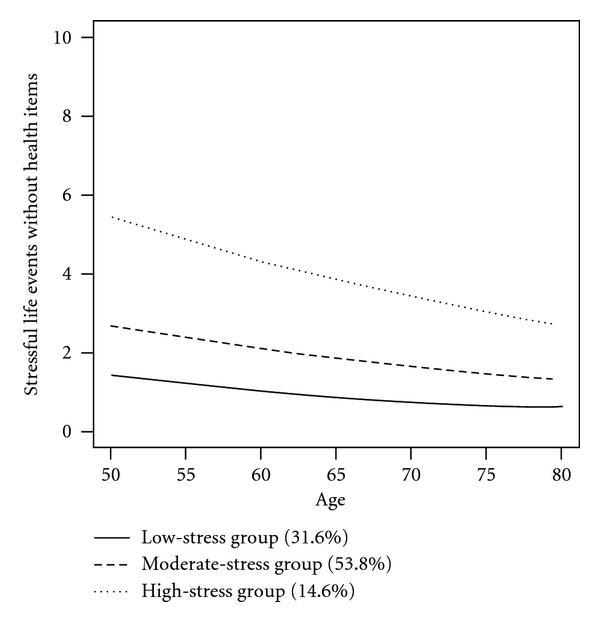
Graphs of the predicted trajectories for the different life event classes (excluding health items).

We repeated the procedure for the life events measure that omitted the two health items. As can be seen in Table 3, only three classes were found for this scoring of the life events measure. Further, none of the quadratic terms were significant, so the final model included only linear terms for the slopes. As can be seen in Figure 3 and Table 4, all of the groups had negative slopes, indicating decreases over time, with low, medium, and high intercepts (Classes 1, 2, and 3 had 32.36%, 55.09%, and 12.54% of the sample, resp.). These class assignments were used in the subsequent analyses predicting mortality, as they omitted the potential confounds of the health items.

### 3.2. Classes of Life Event Trajectories and Mortality

By 2010, 693 (48.02%) of the sample was deceased. The deaths were not equally distributed across the three trajectory classes of stressful life events (no health items), *X* ^2^(2, *N* = 1443) = 15.10, *p* < .01. Only 40.69% of the low-stress group was deceased, compared to 51.19% and 53.04% of the moderate- and high-stress groups, respectively. 

As mentioned earlier, we implemented two different proportional hazards models. In the first model, we investigated the effect of classes of stress trajectories on mortality. Here, we were using the low-stress group as the comparison group. In the second model, we included covariates assessing marital status, education, self-rated health and alcohol and tobacco use. As can be seen in Table 5, both the moderate- and high-stress groups were almost 50% more likely to die than the low-stress group (hazard ratios (95% CI) = 1.43 (1.16, 1.76) and 1.49 (1.10, 2.02), *p*s <.001, and  .01, resp.). Adding the covariates reduced these slightly, but they remained significant (hazard ratios = 1.42 (1.14, 1.76) and 1.37 (1.01, 1.87), *p* = 001 and *p* < .05, resp.). Being married and having better self-reported health were protective factors, while being a nondrinker and being a smoker were risk factors for mortality.

## 4. Discussion

We examined two major issues in the study of aging and stress. First, we examined patterns of change in two measures of stressful life events over 18 years, one a summary score which included health events, and a second that excluded the two health events. Using growth mixture models, we found four patterns for the stressful life event measure which included the health events. Three showed linear decreases over time and were distinguished primarily by the initial number of events (low, moderate, and high stress). The fourth pattern, however, demonstrated a nonlinear inverted U, which peaked at about age 70. In the analysis of the life events measure which excluded health events, the first three patterns were replicated but the fourth pattern was not found, suggesting that the health events accounted for the nonlinearity. 

To our knowledge, this study is unique in two respects: first in examining longitudinal change in self-reported stressors over nearly two decades and second in identifying different patterns of change and relating these patterns to mortality. Earlier studies either spanned much shorter periods of time [10, 11, 16] or used stressful life events coded from interviews [12]. While this latter study found cross-sectional differences between young, middle-aged, and older adults, with younger adults having higher levels of stressful life events, they did not find longitudinal decreases over time. However, our study focused primarily on middle-aged and older men and did find decreases even using a measure which assesses events more likely to occur to older adults. It is possible that a different pattern of results might have occurred had we included young adults or women in the sample. 

Second, we considered whether different patterns of stressful life events were associated with mortality. As reviewed earlier, research has shown a highly variable pattern of results, with different studies showing positive, negative, and no effects on mortality [21, 22, 24, 25]. We hypothesized that multiple assessments of life events to tap stress chronicity might be presumed to show greater effects on health outcomes than a single measure. Indeed, this has long been hypothesized by the allostatic load model [30]. However, studies of allostatic load have been limited by omitting psychosocial measures of chronic stress in favor of clinical measures. Further, some more recent studies failed to find associations between chronic stress and some of the allostatic load biomarkers (see [32], for a review). 

Thus, we sought to conduct a more stringent test of the impact of stress on mortality by examining different patterns of psychosocial stress, which included specific indicators of chronic stress over longer periods of time. Further, we excluded health events, given that some have suggested that the relationship between stress and mortality is limited to health-related events [23]. Thus, we utilized the stress classes generated from the growth mixture models of stressful life events, excluding the ones related to health. Moreover, while most theories of stress and health implicitly assume a linear relationship, we hypothesized that the pattern of results would support a nonlinear model. Specifically, we tested the hormesis model currently being advocated by some biogerontologists, in which moderate stress levels are thought to be protective for health outcomes [6, 7, 34]. 

Using a proportional hazards model, in which the different stress patterns were entered first, and then covariates added (see [54]), we found that both the moderate- and high-stress groups showed significantly higher risk of mortality than the low-stress group. This relationship was only slightly attenuated by the addition of standard health behavior risk factors (e.g., smoking and drinking) as covariates. Thus, our hypotheses were partially supported. Chronic stress did predict mortality even when health-related events were excluded. However, contrary to the prediction from hormesis theory, those with moderate-stress levels did not show enhanced longevity. Rather, the low-stress group showed the lowest levels of mortality. However, it is noteworthy that both the moderate- and high-stress groups showed similar mortality risks, which is also not predicted by the allostatic load model. 

There are a couple of possible explanations for this finding. The first is that the allostatic load model may be largely correct but needs to acknowledge an asymptotic relationship in which higher levels of stress do not confer much additional risk over moderate levels (see [55]). The second possibility reflects the relative youth of the field of hormesis—there are currently few guidelines for what level of stress might be considered hormetic. The low-stress group did have some stress—averaging nearly 2 SLEs during their 50s, dropping down to about 1 in their 80s. It is possible that this low level was actually hormetic and provided protection over the higher levels. In other words, low (but not zero) stress, rather than moderate stress, is protective. One or two stressful life events a year may be manageable, allowing individuals to cope in a fashion which increases mastery, theoretically increasing hormesis. However, anything over two stressful life events might prove overwhelming. To adequately test this, we would need to find populations which experienced no stress, which would be difficult. 

Nonetheless, this nonlinear relationship is quite interesting, and might account for the inconsistency in the findings in the literature. Much as the relationship between age and mental health is muddied by its nonlinear (J-shaped) relationship [56–58], so too the nonlinear, asymptotic relationship between stress and mortality might have created similar inconsistencies. Further research is needed to replicate and test these findings. 

The covariates also showed an interesting pattern of results. Those with better self-rated health showed lower levels of mortality, supporting many other studies [59]. Likewise, married men had lower mortality, as previously found [60]. While education had no significant effect, men who were teetotalers showed higher risk of mortality [61]. Smoking was only marginally related to mortality, undoubtedly because so few of the NAS men have continued to smoke. Nonetheless, stressful life event levels clearly had independent effects on mortality.

### 4.1. Limitations and Future Studies

A major limitation of this study was that the sample consisted primarily of white, middle-class men, and other patterns of results would likely be found in more diverse samples which include women and minorities. It is also possible that other types of stress measures, such as daily stressors, would show a different pattern of results. It is surprising that the effects of life events on mortality did not appear to be mediated through health behavior habits such as drinking and smoking but rather had independent effects. Future studies should examine whether the types of biomarkers used to measure allostatic load mediate the effects of psychosocial stress on mortality. Also, it is possible that the use of longitudinal trajectories may have obscured any hormesis-like relationships. Growth mixture models “smooth out” the curve, and it is possible that an analytical technique which better addresses the intermittent nature of stress might show physiological toughening effects [38].

Nonetheless, this study shows that longitudinal assessments of stressful life events significantly predict mortality independent of standard risk factors and that high stressful life event levels did not convey appreciably greater risk than moderate ones. Future studies should investigate both possible nonlinear relationships between stress and health as well as potential mediating pathways.

## Figures and Tables

**Table 1:** Determining the number of classes for stressful life event trajectories including health items.

Classes	log likelihood	BIC1	AIC	2ΔBIC
1	−15417.42324	−15435.60944	−15422.42324	—
2	−14741.18617	−14773.92133	−14750.18617	1323.38
3	−14634.89249	−14682.17661	−14647.89249	183.49
4	−14602.43102	−14664.26409	−14619.43102	35.83
5	−14590.9149	−14667.29693	−14611.9149	−6.07

**Table 2:** Latent class growth analysis results for stressful life events including health items.

Classes	Parameter	Estimate	Error	Parameter = 0	Prob >|T|
1	Intercept	0.21144	0.05578	3.79	.00
	Linear	−0.03782	0.00476	−7.941	.001

2	Intercept	0.98264	0.03549	27.691	.001
	Linear	−0.02215	0.00221	−10.024	.001

3	Intercept	1.69093	0.02611	64.765	.001
	Linear	−0.02336	0.00202	−11.563	.001

4	Intercept	0.4921	0.31959	1.54	0.1237
	Linear	0.36437	0.06986	5.216	.001
	Quadratic	−0.01735	0.00331	−5.248	.001

	Alpha0	−1.80433	0.05954	−30.302	.001
	Alpha1	0.02563	0.00558	4.598	.001

Group membership					

1	(%)	28.53623	3.29379	8.664	.001
2	(%)	52.28231	2.93452	17.816	.001
3	(%)	18.15619	1.80972	10.033	.001
4	(%)	1.02527	0.37458	2.737	.001

**Table 3:** Determining the number of classes for stressful life events excluding health items.

Classes	log likelihood	BIC1	AIC	2ΔBIC
1	−13698.23027	−13716.41647	−13703.2	—
2	−13147.60309	−13180.33825	−13156.6	1072.16
3	−13061.26944	−13108.55355	−13074.3	143.57
4	−13051.09206	−13112.92514	−13068.1	−8.74

**Table 4:** Latent class growth analysis results for stressful life events including health items.

Classes	Parameter	Estimate	Error	Parameter = 0	Prob >|T|
1	Intercept	−0.01395	0.06973	−0.2	0.8415
	Linear	−0.04067	0.0054	−7.539	.001

2	Intercept	0.838	0.04367	19.187	.001
	Linear	−0.02431	0.0026	−9.335	.001

3	Intercept	1.61165	0.03105	51.905	.001
	Linear	−0.0199	0.00238	−8.363	.001

	Alpha0	−1.52872	0.05863	−26.075	.001
	Alpha1	0.03221	0.00565	5.706	.001

Group membership					

1	(%)	31.61516	3.98985	7.924	.001
2	(%)	53.83532	3.46901	15.519	.001
3	(%)	14.54953	1.66608	8.733	.001

**Table 5:** Proportional hazard models predicting mortality (*N* = 977).

Parameter	Estimate	StdErr	ChiSq	prob	Hazard ratio	HRLower CL	HRUpper CL
*Equation 1*							
Moderate-stress group	0.356	0.107	10.947	.001	1.428	1.156	1.764
High-stress group	0.398	0.155	6.638	.010	1.490	1.100	2.018

*Equation 2*							
Moderate-stress group	0.352	0.109	10.440	.001	1.422	1.149	1.761
High-stress group	0.315	0.157	4.023	.045	1.371	1.007	1.866
Health rating	− 0.288	0.064	20.497	.001	0.749	0.661	0.849
Married	− 0.331	0.131	6.458	.011	0.718	0.556	0.927
Less than high school	− 0.231	0.184	1.596	.207	0.793	0.553	1.136
College	− 0.121	0.111	1.174	.279	0.886	0.712	1.103
Postgrad	0.038	0.150	0.065	0.799	1.039	0.774	1.394
Nondrinker	0.314	0.123	6.496	0.011	1.369	1.075	1.743
Heavy drinker	− 0.009	0.125	0.005	0.944	0.991	0.776	1.267
Smoker	0.215	0.127	2.859	0.091	1.240	0.966	1.591

## References

[1] Aldwin C, Stokols D (1988). The effects of environmental change on individuals and groups: some neglected issues in stress research. *Journal of Environmental Psychology*.

[2] Elder G, Shanahan M, Lerner R, Damon W (2006). The life course and human development. *Handbook of Child Psychology*.

[3] Dannefer D, Birren JE, Bengtson VL (1988). What’s in a name? An account of the neglect of variability in the study of aging. *Emergent Theories of Aging*.

[4] McEwen BS (2005). Glucocorticoids, depression, and mood disorders: structural remodeling in the brain. *Metabolism: Clinical and Experimental*.

[5] Calabrese EJ (2005). Paradigm lost, paradigm found: the re-emergence of hormesis as a fundamental dose response model in the toxicological sciences. *Environmental Pollution*.

[6] Calabrese EJ, Le Bourg E, Rattan SIS (2008). What is hormesis?. *Mild Stress and Healthy Aging: Applying Hormesis in Aging Research and Interventions*.

[7] Lithgow GJ, White TM, Melov A, Johnson TE (1995). Thermotolerance and extended life-span conferred by single-gene mutations and induced by thermal stress. *Proceedings of the National Academy of Sciences of the United States of America*.

[8] Aldwin C, Stephens MAP, Crowther JH, Hobfoll SE, Tennenbaum DL (1990). The elders life stress inventory (ELSI): egocentric and nonegocentric stress. *Stress and Coping in Late Life Families*.

[9] Aldwin C (1991). Does age affect the stress and coping process? The implications of age differences in perceived locus of control. *The Journals of Gerontology B*.

[10] George LK, Lynch SM (2003). Race differences in depressive symptoms: a dynamic perspective on stress exposure and vulnerability. *Journal of Health and Social Behavior*.

[11] Lynch SM, George LK (2002). Interlocking trajectories of loss-related events and depressive symptoms among elders. *Journals of Gerontology B*.

[12] Chiriboga DA, Lachman ME, James JB (1997). Crisis, challenge, and stability in the middle years. *Multiple Paths of Midlife Development*.

[13] Yancura LA, Aldwin CM, Spiro A (1999). Does stress decrease with age? A longitudinal examination of stress in the normative aging study. *The Gerontologist*.

[14] Ensel WM, Lin N (1996). Distal stressors and the life stress process. *Journal of Community Psychology*.

[15] Ensel WM, Lin N (2000). Age, the stress process, and physical distress: the role of distal stressors. *Journal of Aging and Health*.

[16] Lorenz FO, Simons RL, Conger RD, Elder GH, Johnson C, Chao W (1997). Married and recently divorced mothers’ stressful events and distress: tracing change across time. *Journal of Marriage and Family*.

[17] Cohen S, Janicki-Deverts D, Miller G (2007). Psychological stress and disease. *Journal of the American Medical Association*.

[18] Krantz DS, McCeney MK (2002). Effects of psychological and social factors on organic disease: a critical assessment of research on coronary heart disease. *Annual Review of Psychology*.

[19] Ramachandruni S, Handberg E, Sheps DS (2004). Acute and chronic psychological stress in coronary disease. *Current Opinion in Cardiology*.

[20] Singer BH, Ryff CD (2001). *New Horizons in Health: An Integrative Approach*.

[21] Lantz PM, House JS, Mero RP, Williams DR (2005). Stress, life events, and socioeconomic disparities in health: results from the Americans’ changing lives study. *Journal of Health and Social Behavior*.

[22] Rosengren A, Orth-Gomer K, Wedel H, Wilhelmsen L (1993). Stressful life events, social support, and mortality in men born in 1933. *British Medical Journal*.

[23] Phillips AC, Der G, Carroll D (2008). Stressful life-events exposure is associated with 17-year mortality, but it is health-related events that prove predictive. *British Journal of Health Psychology*.

[24] Maunsell E, Brisson J, Mondor M, Verreault R, Deschênes L (2001). Stressful life events and survival after breast cancer. *Psychosomatic Medicine*.

[25] Hollis JF, Connett JE, Stevens VJ, Greenlick MR (1990). Stressful life events, type A behavior, and the prediction of cardiovascular and total mortality over six years. *Journal of Behavioral Medicine*.

[26] Kopp MS, Rethelyi J (2004). Where psychology meets physiology: chronic stress and premature mortality—the Central-Eastern European health paradox. *Brain Research Bulletin*.

[27] Pearlin LI, Aneshensel CS, Mullan JT, Whitlatch CJ, Binstock RH, George LK (1996). Caregiving and its social support. *Handbook of Aging and the Social Science*.

[28] Schulz R, Beach SR (1999). Caregiving as a risk factor for mortality: the caregiver health effects study. *Journal of the American Medical Association*.

[29] Fredman L, Cauley JA, Hochberg M, Ensrud KE, Doros G (2010). Mortality associated with caregiving, general stress, and caregiving-related stress in elderly women: Results of caregiver-study of osteoporotic fractures. *Journal of The American Geriatrics Society*.

[30] McEwen BS, Seeman T (1999). Protective and damaging effects of mediators of stress: elaborating and testing the concepts of allostasis and allostatic load. *Annals of the New York Academy of Sciences*.

[31] Juster R, McEwen BS, Lupien SJ (2010). Allostatic load biomarkers of chronic stress and impact on health and cognition. *Neuroscience and Biobehavioral Reviews*.

[32] Gersten O, Dow WD, Rosero-Bixby L (2010). Stressors over the life course and neuroendocrine system dysregulation in Costa Rica. *Journal of Aging and Health*.

[33] Yancura LA, Aldwin CM, Levenson MR, Spiro A (2006). Coping, affect, and the metabolic syndrome in older men: how does coping get under the skin?. *Journals of Gerontology B*.

[34] Le Bourg E, Rattan SIS, Le Bourg E, Rattan SIS (2008). Hormesis and aging: what’s the deal?. *Mild Stress and Healthy Aging: Applying Hormesis in Aging Research and Interventions*.

[35] Vaiserman AM, Le Bourg E, Rattan SIS (2008). Irradiation and hormesis. *Mild stress and Healthy Aging: Applying Hormesis in Aging Research and Interventions*.

[36] Chiueh C, Andoh T, Chock PB (2005). Induction of thioredoxin and mitochondrial survival proteins mediates preconditioning-induced cardioprotection and neuroprotection. *Annals of the New York Academy of Sciences*.

[37] Holzman D (1995). Hormesis: fact or fiction?. *Journal of Nuclear Medicine*.

[38] Dienstbier RA (1989). Arousal and physiological toughness: implications for mental and physical health. *Psychological Review*.

[39] Calhoun L, Tedeschi R, Calhoun L, Tedeschi R (2006). The foundations of posttraumatic growth. *The Handbook of Posttraumatic Growth: Research and Practice*.

[40] Schnurr P, Rosenberg S, Friedman M (1993). Change in MMPI scores from college to adulthood as a function of military service. *Journal of Abnormal Psychology*.

[41] Hobfoll SE (2002). Social and psychological resources and adaptation. *Review of General Psychology*.

[42] Vaillant G (2002). *Aging Well: Surprising Guideposts to a Happier Life*.

[43] Jones BL, Nagin DS, Roeder K (2001). A SAS procedure based on mixture models for estimating developmental trajectories. *Sociological Methods and Research*.

[44] Spiro A, Bosse’ R, Maddox G (2001). The Normative Aging Study. *Encyclopedia of Aging*.

[45] Aldwin C, Levenson MR, Spiro A, Bossé R (1989). Does emotionality predict stress? Findings from the normative aging study. *Journal of Personality and Social Psychology*.

[46] Ware JE, Sherbourne CD (1992). The MOS 36-item short-form health survey (SF-36). I. Conceptual framework and item selection. *Medical Care*.

[47] Nagin DS (1999). Analyzing developmental trajectories: a semiparametric, group-based approach. *Psychological Methods*.

[48] Lambert D (1992). Zero-inflated poisson regression, with an application to defects in manufacturing. *Technometrics*.

[49] Vuong Q (1989). Likelihood ratio tests for model selection and non-nested hypotheses. *Econometrica*.

[50] Schwartz G (1978). Estimating the dimension of a model. *The Annals of Statistics*.

[51] Kass RE, Wasserman L (1995). A reference Bayesian test for nested hypotheses and its relationship to the Schwarz criterion. *Journal of the American Statistical Association*.

[52] D’Unger AV, Land KC, McCall PL, Nagin DS (1998). How many latent classes of delinquent/criminal careers? Results from mixed Poisson regression analyses of the London, Philadelphia, and Racine Cohorts studies. *The American Journal of Sociology*.

[53] Cox DR (1972). Regression models and life tables (with discussion). *Journal of the Royal Statistical Society B*.

[54] Mroczek DK, Spiro A, Turiano NA (2009). Do health behaviors explain the effect of neuroticism on mortality? Longitudinal findings from the VA normative aging study. *Journal of Research in Personality*.

[55] Rutter M (1989). Pathways from childhood to adult life. *Journal of Child Psychology and Psychiatry and Allied Disciplines*.

[56] Aldwin C, Spiro A, Levenson MR, Bossé R (1989). Longitudinal findings from the normative aging study: I. Does mental health change with age?. *Psychology and Aging*.

[57] Newman J (1989). Aging and depression. *Psychology and Aging*.

[58] Kessler RC, Foster C, Webster PS, House JS (1992). The relationship between age and depressive symptoms in two national surveys. *Psychology and Aging*.

[59] Idler EL, Benyamini Y (1997). Self-rated health and mortality: a review of twenty-seven community studies. *Journal of Health and Social Behavior*.

[60] Tucker JS, Friedman HS, Wingard DL, Schwartz JE (1996). Marital history at midlife as a predictor of longevity: alternative explanations to the protective effect of marriage. *Health Psychology*.

[61] Holahan CJ, Schutte KK, Brennan PL, Holahan CK, Moos BS, Moos RH (2010). Late-life alcohol consumption and 20-year mortality. *Alcoholism: Clinical & Experimental Research*.

